# SHANK3 Antibody Validation: Differential Performance in Western Blotting, Immunocyto- and Immunohistochemistry

**DOI:** 10.3389/fnsyn.2022.890231

**Published:** 2022-06-06

**Authors:** Anne-Kathrin Lutz, Helen Friedericke Bauer, Valentin Ioannidis, Michael Schön, Tobias M. Boeckers

**Affiliations:** ^1^Institute for Anatomy and Cell Biology, Ulm University, Ulm, Germany; ^2^Deutsches Zentrum für Neurodegenerative Erkrankungen (DZNE), Ulm, Germany

**Keywords:** SHANK3, Western Blot, autism, ASD, immunohistochemistry, specificity

## Abstract

SHANK3 is a scaffolding protein implicated in autism spectrum disorders (ASD). Its function at excitatory glutamatergic synapses has been studied for the last two decades, however, tissue-specific expression patterns as well as its subcellular localization need to be studied in further detail. Especially the close sequence homology of SHANK3 to its protein family members SHANK2 and SHANK1 raises the emerging need for specific antibodies that are validated for the desired methodology. With this study, we aim to validate a set of commercial as well as homemade SHANK3 antibodies in Western Blotting, and synaptic immunocyto- and immunohistochemistry. We found that only a small subset of the antibodies included in this study meet the criteria of quality and specificity. Therefore, we aim to share our findings on SHANK3 antibody validation but also raise awareness of the necessity of antibody specificity testing in the field.

## Introduction

The SHANK3 protein, also known as ProSAP2, is an important scaffolding protein for the post-synaptic specialization of glutamatergic synapses in the central nervous system (Boeckers et al., 1999, 2002; Naisbitt et al., 1999; Grabrucker et al., 2011b). There, it provides an anchoring platform for surface protein receptor molecules, including AMPARs, NMDARs, and mGluRs, and links them to the actin cytoskeleton (Naisbitt et al., 1999; Tu et al., 1999; Jiang and Ehlers, 2013). During neurodevelopment, SHANK3 is important for proper dendritic spine formation but also spine maturation and maintenance (Sala et al., 2001; Grabrucker et al., 2011a). The linkage to the actin cytoskeleton propagates signal-dependent neuronal plasticity, which is important for learning and strengthening synaptic connections. Besides the central nervous system as its best-known localization and expression, SHANK3 is also expressed in various other tissues such as the skeletal muscle (Lutz et al., 2020) and the gastrointestinal system (Pfaender et al., 2017; Sauer et al., 2019). The sheer abundance of SHANK3 highlights the importance to study the precise molecular functions of SHANK3 in each of these tissues.

SHANK3 deficiency is implicated in autism spectrum disorders (ASD; Durand et al., 2007). More specifically, heterozygous deletions or mutations in the distal section of the long arm of chromosome 22, where the SHANK3 gene locates, lead to Phelan–McDermid syndrome (PMDS) also known as 22q13.3 deletion syndrome (Phelan, 2008; Phelan and McDermid, 2012). The loss of SHANK3 has detrimental effects manifesting in global developmental delay, intellectual disability, autism-like behavior, and muscular neonatal hypotonia in affected individuals. This highly impacts the quality of daily life of both patients with PMDS but also their families and caretakers (Phelan, 2008; Phelan and McDermid, 2012; Phelan et al., 2022).

To study the consequences of SHANK3 deficiency on a molecular level, various model systems have been employed. Knock-down experiments in primary neurons (Verpelli et al., 2011) and human-induced pluripotent stem cell–derived cells including enterocytes (Pfaender et al., 2017), neurons (Shcheglovitov et al., 2013; Lutz et al., 2020), and muscle cells (Lutz et al., 2020) have been performed and various mouse models have been generated carrying different deletions and mutations of SHANK3 (Jiang and Ehlers, 2013; Delling and Boeckers, 2021). Interestingly, these SHANK3-deficient mouse models manifest with different phenotypes dependent on the location of the deletion in *Shank3* (Delling and Boeckers, 2021). Dependent on multiple internal promotors, six different isoforms of SHANK3 are known, and with alternative splicing at least 10 SHANK3 isoforms can be generated (Wang et al., 2014). These isoforms are brain-region as well as cell-type specific (Wang et al., 2014). To elucidate the role of SHANK3, the tissue-specific and the subcellular localization need to be studied in an appropriate, reliable, and reproducible way. None of the SHANK3 isoforms contains a unique amino acid sequence that would allow for an isoform-specific antibody production. On the other hand, this means that each SHANK3 antibody will always detect multiple isoforms, however, no antibody can detect all isoforms at once. Therefore, it is crucial to assess the respective antigen before using a SHANK3 antibody. In addition, since SHANK3 has a high-sequence homology with both SHANK1 and SHANK2, the other two members of the SHANK protein family, it is of special importance to have antibodies in hands that specifically recognize SHANK3.

To provide evidence for SHANK3 antibody validity, we designed this study that combines three main methodologies to test SHANK3 antibodies. First, Western Blotting was performed on mouse cortical wild-type (WT) and *Shank3*Δ*ex11(–/–)* (KO) tissue lysate to assess the reactivity with SHANK3-specific isoforms. In addition, SHANK3 specificity was assessed by testing for cross-reactivity with overexpressed SHANK1 and SHANK2 protein. Second, antibodies were examined on their performance in immunocytochemistry (ICC) of rat primary hippocampal neurons, and third, brain sections of WT and KO mice were stained immunohistochemically (IHC). Six commercially available and three homemade SHANK3 antibodies, obtained from different species, were included in this study. We found that following standard procedures for Western Blotting, ICC, and IHC, certain antibodies were not SHANK3-specific or only worked in a certain method. Therefore, we want to raise the awareness for the necessity of good antibody validation. In this study, we defined specific SHANK3 antibodies that can reliably be used in Western Blotting, ICC, or IHC experiments.

## Materials and Methods

### Animal Ethics Statement

*Shank3*Δ*11(–/–)* mice were previously described (Schmeisser et al., 2012). All mice were housed in standard laboratory conditions with food and water *ad libitum*, an average temperature of 22°C and a 12 h dark/light cycle. All animal experiments were conducted in compliance with the guidelines for the welfare of experimental animals issued by the Federal Government of Germany, ID numbers O.103 and 1497.

### Antibody Production

**FIGURE 1:**
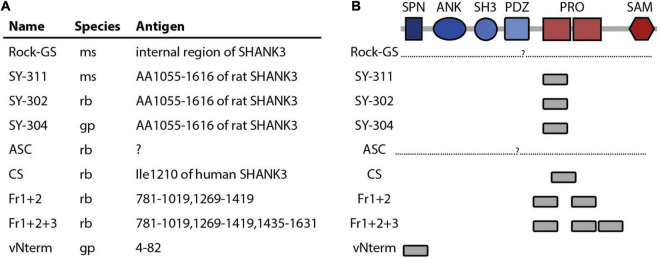
SHANK3 antibodies used in this study. **(A)** Name, company, catalog number, species and, if know, the antigen of the nine SHANK3 antibodies used in this study are listed. **(B)** The protein domains of SHANK3 are given and the location of the antibody binding is indicated. SPN (Shank/ProSAP N-terminal domain), ANK (ankyrin repeat domain), SH3 (src Homology-3), PDZ (PSD-95/DLG/ZO-1 domain), PRO (proline rich domain), SAM (sterile alpha motif domain).

Homemade SHANK3 antibodies were produced as follows. *Fr1*+*2*: This antibody has been published before and its production has been described in detail (Schmeisser et al., 2012). *Fr1*+*2+3*: This antibody has been produced in parallel to Fr1+2 using the same methodology. Fragments of SHANK3 are given in Figure 1A. *vNterm*: First, a 234 bp long fragment from the N-terminus of *Shank3* was amplified by PCR (template: homemade rat-sequence *Shank3* DNA plasmid in a pGEX-4T-1 vector; see following sequence, primer sequences underlined).

aaccgtgccgccgtcgccgccgccgctgcgcctgcggagcccccggagccgctgtcccc cgcgctggccccggccccggccccccccggccccctcccgcgtagcgcggtcggcgggact ctggcggggggtcagggggggccagggcgccgcgcggagtccccgtgcgctcctctctccg ccgggaacagtcagggccccggcgctagcaccgggatggacggccccggggcc

An *Eco*RI was incorporated into the forward primer, and an *Xho*I restriction site was incorporated into the reverse primer, and *via* these, into an inducible expression vector (pGEX-4T-1). This was subsequently transformed into *Escherichia coli* BL21 bacteria. Expression was subsequently stimulated with IPTG. After successful small-scale expression (verified by SDS-PAGE), large-scale overexpression was performed. After lysis, the suspension was added to columns containing glutathione agarose. GST–SHANK3 fusion proteins were able to bind to this. Subsequently, elution was performed *via* glutathione. The purified peptide was then sent to Pineda Antibody Service (Berlin, Germany), where various animals were immunized (including rabbits). The sera of the final bleeding were finally frozen at –80°C.

### Cell Culture and Transfection

Full-length rat GFP-Shank1A was already described (Romorini et al., 2004), as well as full-length rat GFP-tagged Shank2 (Boeckers et al., 2005) and full-length rat GFP-Shank3a (Grabrucker et al., 2011a). HEK293T cells were cultivated in DMEM (Gibco) supplemented with 10% fetal bovine serum (FBS, Gibco) at 37°C in 5% CO_2_. For transient transfection, cells were plated on 10-cm petri dishes and cultivated until 70–80% confluence and were then transfected with either full-length rat GFP-Shank1A, rat GFP-tagged Shank2 or rat GFP-Shank3a. Therefore, an appropriate amount of DNA was mixed with DMEM with 10% FBS and PEI MAX 40K (Polysciences) transfection reagent and spread onto the cells. The cells were lysed 24 h after the transfection (lysis buffer of the μMACS GFP Isolation Kit (Miltenyi Biotec) + PhosphoSTOP and Complete Mini EDTA-free Protease Inhibitor Cocktail (both Roche) and purification of the recombinant GFP-tagged SHANK1, 2, and 3 proteins was performed using the μMACS GFP Isolation Kit (Miltenyi Biotec) according to manufacturer’s instructions. Purified recombinant proteins were stored at –20°C until usage.

### Western Blotting

Homogenates of cortices from *Shank3(*+*/*+) and *Shank3*Δ*ex11(–/–)* mice were used. Therefore, animals were decapitated after deep CO_2_ narcosis, the cortex was dissected, and the tissue was homogenized in 0.32 M sucrose (Carl Roth), 5 mM HEPES (Carl Roth) pH 7.4, PhosphoSTOP, and Complete Mini EDTA-free Protease Inhibitor Cocktail (both Roche) using a Teflon douncer (Sartorius) and Potter S (B. Braun Biotech International) with 12 strokes at 900 rpm. Protein concentrations of homogenates were determined by Bradford assay. Samples were diluted with 4 × sodium dodecyl sulfate (SDS) loading buffer (200 mM Tris–HCl (AppliChem), pH 6.8, 200 mM dithiothreitol (Sigma-Aldrich), 4% SDS (Carl Roth), 40% glycerol (Sigma-Aldrich), and 0.02% bromphenol blue (Sigma-Aldrich). For Western Blot analysis, equal amounts of 5 μg total protein were separated with polyacrylamide gel electrophoresis (SDS-PAGE) and blotted on nitrocellulose membranes with the Trans-Blot Turbo Transfer System (BioRad). Membranes were blocked with blocking solution (5% bovine serum albumin (BioFROXX), in Tris-buffered saline with 0.1% Tween-20 (Carl Roth; TBST 0.1%), and primary antibodies were incubated in blocking solution overnight at 4°C. The primary antibodies are listed in Table 1.

**TABLE 1:** Primary antibodies.

Antibody	Company, Cat No	WB	IHC	ICC
β-ACTIN ms	Sigma, A5316	1:250,000	–	–
GFP Living Colors A.v. Monoclonal Antibody (JL-8)	Takara, 632381	1:1000	–	–
SYNAPSIN rb	SYSY, 106 003	–	1:1000	1:1000
BASSOON ms	Enzo, ADI-VAM-PS003	–	1:1000	1:500
MAP2	EnCor, CPCA-MAP2	–	–	1:1000
SHANK3 ms (Rock-GS)	Rockland, 200-301-GS	1:1000	1:500	1:500
SHANK3 ms (SY-311)	SYSY, 162 311	1:1000	1:500	1:500
SHANK3 rb (SY-302)	SYSY, 162 302	1:1000	1:500	1:500
SHANK3 gp (SY-304)	SYSY, 162 304	1:1000	1:500	1:1000
SHANK3 rb (ASC)	Abgent, ASC11481	1:1000	1:500	1:500
SHANK3 rb (CS)	Cell Signaling, 64555S	1:1000	1:500	1:200
SHANK3 rb (Fr1+2)	Homemade	1:1000	1:2000	1:500
SHANK3 rb (Fr1+2+3)	Homemade	1:1000	1:1000	1:500
SHANK3 gp (vNterm)	Homemade	1:1000	1:1000	1:500

Subsequently, membranes were washed with TBST 0.2%, incubated with horseradish peroxidase (HRP)–conjugated secondary antibodies (Dako) and visualized with ECL substrate (Thermo Scientific) in a MicroChemi 4.2 station (BNC Bio-Imaging Systems).

### Immunohistochemistry

For IHC, *Shank3(*+*/*+) and *Shank3*Δ*ex11(–/–)* mice were anesthetized with 20 μl/g bodyweight (25% (WDT, 10%), 5% Xylazine (Rompun 2%, Bayer) dissolved in 0.9% NaCl solution (Sigma-Aldrich). Then they were perfused with 25 ml cooled PBS without calcium and magnesium (PBS–/–, Gibco) followed by 50 ml 4% paraformaldehyde (PFA). Brains were dissected, post-fixated overnight with 4% PFA at 4°C and subsequently immersed in 30% sucrose. Then, brains were frozen in Tissue-Tek O.C.T. Compound (Sakura Finetek) on dry ice and stored at –80°C until sectioning.

Brains were sectioned at 30 μm at a cryostat (Leica CM1950) and collected as free-floating sections in PBS–/–. For storage, they were submerged in 50% PBS–/–, 30% ethylene glycol, and 20% glycerol and stored at –20°C. Before usage, they were washed three times in PBS–/–, and then blocked and permeabilized in 5% FBS, 0.2% Triton for 4 h. Primary antibodies were diluted in blocking solution and incubated at 4°C for 48 h (Table 1). The primaries were washed four times with PBS+/+ and secondary antibodies produced in donkey and coupled to Alexa Fluor 488, 594, or 647 (Jackson Laboratory) were incubated for 2 h at RT in blocking solution. After washing again with PBS+/+ tissue was mounted with VectaMount (Vector) with DAPI (1:50,000). Sections were imaged using a laser-scanning microscope (Leica DMi8). Images were captured with a 40 × oil DIC immersion objective using the LasX software (Leica), with a resolution of 2,048 × 2,048 pixels.

### Immunocytochemistry

Primary rat hippocampal neurons were prepared and plated on cover slips following standard procedures and as published elsewhere (Grabrucker et al., 2009). At DIV14, cells were fixed using 4% paraformaldehyde (Merck) with 4% sucrose (Roth) in DPBS (Gibco) at 37°C for 15 min. Neurons were washed 3 × with PBS+/+ that included Ca^2+^ and Mg^2+^ (PAA) and permeabilized with 0.2% Triton-X100 (Roche) in PBS+/+ for 10 min. Cells were blocked in 5% fetal bovine serum (Gibco) and 10% donkey serum (Millipore) in PBS++ for 2–4 h. Primary antibodies (Table 1) were incubated at 4°C for 24 h in a blocking solution. The primaries were washed three times with PBS+/+ and secondary antibodies produced in donkey and coupled to Alexa Fluor 488, 594, or 647 (Jackson Laboratory) were incubated for 1 h at RT in blocking solution. Cells were washed again and then mounted with ProLong Gold antifade reagent with DAPI (Thermo Fisher Scientific). Fluorescent images were recorded using an Axioscope microscope with a Zeiss CCD camera (16 bits; 1,280 × 1,024 ppi) and Axiovision software (Zeiss).

### Image Analysis and Quantification

Pictures of rat primary hippocampal neurons were deconvoluted using Autoquant X3 Deconvolution software (Imaris). Synaptic puncta along 30 μm of secondary dendrites were determined using ImageJ and the “FindFoci” plugin (Herbert et al., 2014). Mouse brain sections between bregma –1.15 and –1.955 have been used for analysis. The cortical analysis was exclusively performed in the MOs, the secondary motor cortex. In the confocal pictures of mouse brain sections, regions of interest (ROIs) of 17 μm^2^ were chosen. ROIs were used for FindFoci analysis as well.

Colocalization was performed in R with a custom script. In brief: For each SHANK3 puncta in an ROI the Euclidean distance to each non-SHANK3 puncta was measured by raster::pointDistance() function. If the distance to non-SHANK3 puncta was smaller than 0.75 μm, the SHANK3 puncta were classified as colocalizing.

Data were ether grouped by replicate and SHANK3 antibody for the primary neuron experiments or by replicate, SHANK3 antibody, and brain region for the brain section experiments. All variables were then normalized on the mean of colocalizing puncta’s values (in wild-type) for the respective variable. Mean values of normalized data were calculated for each neuron or animal’s brain region for each variable, respectively.

Count: All puncta stained by the respective SHANK3 antibody of all pictures, all experiments, and all ROIs were considered. Relative count in percentage: The count was set to 100% and the number of co-localizing and not co-localizing puncta was calculated and depicted.

Further analysis and plotting were done using R version 4.1.0. Following packages were used: tidyverse, stringi, openxlsx, rstatix, ggplot, and raster.

### Statistical Analysis

For statistical comparison of colocalization classification in primary neurons, data was group-wise tested for normal distribution using rstatix::shapiro_test() first. Normality was rejected in at least one group of all comparisons, therefore rstatix::wilcox_test() was used for hypothesis testing.

Statistical comparison of genotype and colocalization classification in brain regions was done using rstatix::anova_test with standard settings and the following linear model:


$$\text{variable}∼{\text{genotype}}^{*}\,\mathrm{colocClass}$$

The main effects of genotype and colocalization classification as well as genotype-colocalization interaction were reported. Significance value was set to 0.05 with *p ≤ 0.05, ^**^p ≤ 0.01, ^***^p ≤ 0.001, and ^****^p ≤ 0.0001.

## Results

### Employed SHANK3 Antibodies and Their Epitopes

The nine antibodies used in this study are summarized in Figure 1A and listed in Table 1. Two antibodies have been produced in mouse (ms), two in guineapig (gp) and five in rabbit (rb). Six antibodies are commercially available and three are homemade. For two antibodies (Rock-GS and ASC), the exact binding region is unknown and not provided by the manufacturer (Figure 1B). The three antibodies from Synaptic Systems are all directed against the same antigen. Most antibodies bind to the Proline-rich region (PRO) and the only antibody targeting the N-terminus of SHANK3 is the homemade vNterm (very N-terminal) antibody. The homemade antibodies Fr1+2 and Fr1+2+3 share fragment 1 and fragment 2 for antibody binding.

### Not All Antibodies Specifically Detect SHANK3 in Western Blotting

**FIGURE 2:**
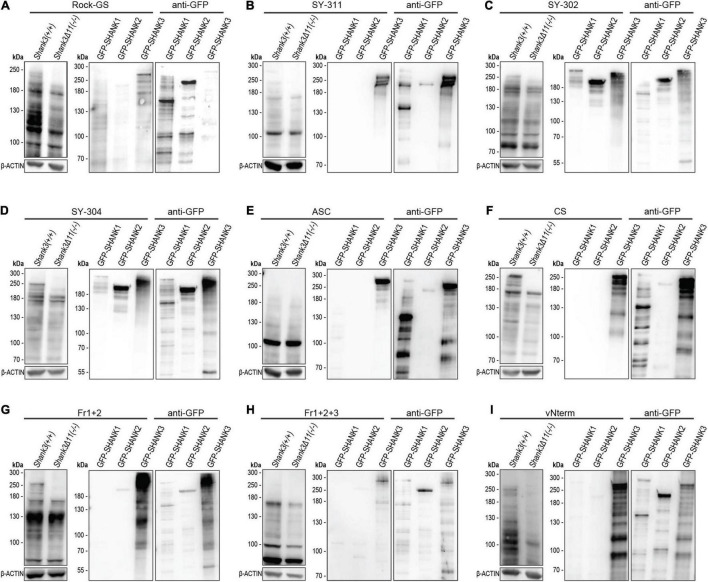
Western Blot analysis for SHANK3 specificity. **(A)** Rock-GS, **(B)** SY-311, **(C)** SY-302, **(D)** SY-304, **(E)** ASC, **(F)** CS, **(G)** Fr1+2, **(H)** Fr1+2+3, and **(I)** vNterm. Left: The Western Blot shows mouse cortical lysate of WT and KO mice for all antibodies. Same amounts of protein are ensured by β-ACTIN. Right: GFP-SHANK1, GFP-SHANK2, and GFP-SHANK3 were overexpressed in HEK293T cells. Blot were first incubated with the respective SHANK3 antibody and then with an anti-GFP antibody to ensure plasmid expression.

First, we examined if the SHANK3 antibodies were able to detect the SHANK3 loss in cortical lysate of Shank3 KO animals (Figures 2A–I). This analysis is shown in the left part of each antibody figure and equal loading was ensured by β-ACTIN. A loss of the largest two SHANK3 isoforms was observed with the Rock-GS, SY-311, SY-302, SY-304, CS, Fr1+2, and vNterm SHANK3 antibodies. The ASC antibody and the Fr1+2+3 antibody did not detect the SHANK3 isoforms larger than 180 kDa. The ASC did not show any change in signal in the KO, the Fr1+2+3, however, did show a decreased SHANK3 signal in the KO tissue in the detected isoforms. All blots have been repeated in independent experiments. For those antibodies, that did not give a clear signal, the blots were repeated to exclude a technical artifact. Next, to check if the antibodies react SHANK3-specific or if they are cross-reactive with SHANK1 and SHANK2, GFP-SHANK1, GFP-SHANK2, and GFP-SHANK3 fusion proteins were overexpressed in HEK293T cells that do not express SHANKs endogenously. Correct overexpression was ascertained by GFP antibody (right part of each membrane). Cross-reactivity with SHANK1 and SHANK2 was observed for Rock-GS, SY-302, and SY-304. These three antibodies are, therefore, based on our experiments, not suited for any SHANK3-specific analysis. Despite a minor background stain in the SHANK2 lane for Fr1+2 and vNterm, SHANK3 antibodies that are both SHANK3 specific and can differentiate between WT and *Shank3* KO material are SY-311, CS, Fr1+2, and vNterm.

### Only Some SHANK3 Antibodies Display a Specific Synaptic SHANK3 Staining in Primary Rat Hippocampal Neurons

**FIGURE 3:**
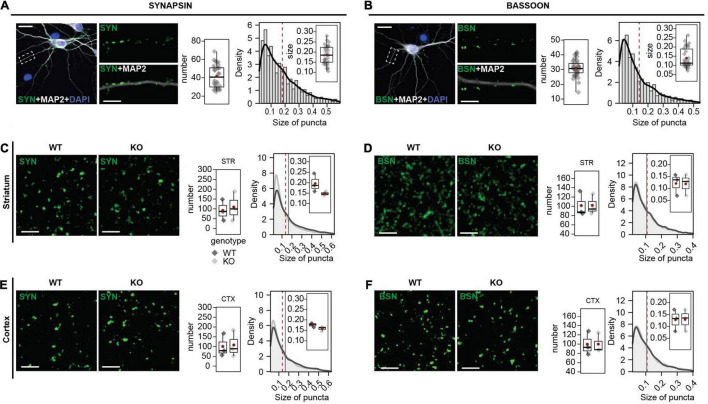
SYNAPSIN1/2 and BASSOON expression in primary hippocampal neurons, mouse striatum, and cortex. **(A)** SYNAPSIN1/2 and MAP2 ICC in primary hippocampal neurons. The number of SYNAPSIN1/2 positive synapses per 30 μm secondary dendrite and the size of the puncta were analyzed. **(B)** BASSOON and MAP2 ICC in primary hippocampal neurons. The number of BASSOON-positive synapses per 30 μm secondary dendrite and the size of the puncta were analyzed. Scale bar 20 μm. **(C)** SYNAPSIN1/2 IHC in striatum of WT and *Shank3* KO mice. The number of SYNAPSIN1/2-positive synapses per 17 μm^2^ and the size of the puncta were analyzed. Scale bar 20 μm. **(D)** BASSOON IHC in striatum of WT and *Shank3* KO mice. The number of BASSOON-positive synapses per 17 μm^2^ and the size of the puncta were analyzed. **(E)** SYNAPSIN1/2 IHC cortex of WT and *Shank3* KO mice. The number of SYNAPSIN1/2-positive synapses per 17 μm^2^ and the size of the puncta were analyzed. **(F)** BASSOON IHC in cortex of WT and *Shank3* KO mice. The number of BASSOON-positive synapses per 17 μm^2^ and the size of the puncta were analyzed. Scale bar 5 μm. Left graph: Number of synapses. Right graph: Size of SYNAPSIN1/2 puncta, frequency distribution (bins) and estimated density (black line). The boxplot shows the median and the interquartile range, the whiskers cover minimal-to-maximal values. The red dot or the red line mark the mean. ICC: Data collected from a total of 40 cells of 4 independent experiments. IHC: *n* = 3 mice per genotype. WT: *Shank3(*+*/*+), KO: *Shank3*Δ*ex11(–/–)* mice. Groups were tested for normality using the Shapiro–Wilk test and then compared using the Wilcoxon test.

For this study, we aimed to validate antibodies that show a specific synaptic SHANK3 expression. SHANK3 is also expressed extra-synaptically (Durand et al., 2012; Grabrucker et al., 2014) and the potential expression of different SHANK3 isoforms in different sub-cellular localizations could further contribute to a SHANK3 expression that is not restricted to synapses. However, synaptic SHANK3 localization is well described (Grabrucker et al., 2011b,^2014^) and of great importance for further research on synaptic malfunction under SHANK3 deficiency. The different SHANK3 antibodies were co-stained with SYNAPSIN or BASSOON as pre-synaptic markers. In respect of the species of the SHANK3 antibodies, SHANK3-ms antibodies were co-stained with SYNAPSIN-rb, and all others with BASSOON-ms. First, the number and size of the SYNAPSIN (Figure 3A) and BASSOON puncta (Figure 3B) were analyzed in rat hippocampal neurons. This analysis was performed to ensure reproducibility between our experiments. In mouse brain sections, we analyzed SYNAPSIN1/2 (Figure 3C) and BASSOON in striatum (STR; Figure 3D). As a second region, we analyzed SYNAPSIN1/2 (Figure 3E) and BASSON (Figure 3F) in the cortex (CTX). The number and the size of synaptic puncta were found to be the same in WT and KO mice.

**FIGURE 4:**
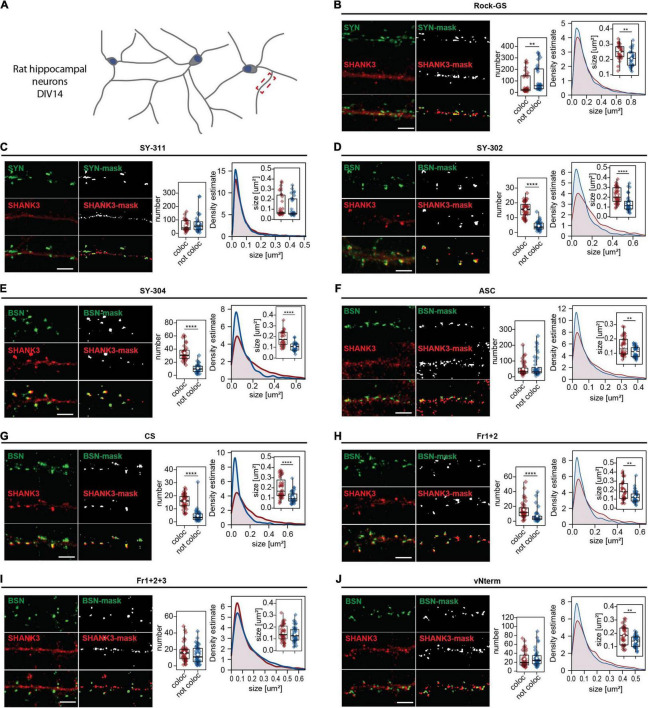
ICC analysis in primary rat hippocampal neurons. **(A)** Neurons have been fixed at DIV14 and secondary dendrites were analyzed. Cells were stained against MAP2, SYNAPSIN1/2, or BASSOON and **(B)** Rock-GS, **(C)** SY-311, **(D)** SY-302, **(E)** SY-304, **(F)** ASC, **(G)** CS, **(H)** Fr1+2, **(I)** Fr1+2+3, and **(J)** vNterm. Scale bar 5 μm. ICC and the derived mask obtained in ImageJ with FindFoci are shown. Left graph: Number of synapses. SHANK3 puncta co-localizing with the respective pre-synaptic marker (red) or not co-localizing (blue) are shown. Right graph: Size of SHANK3 puncta and estimated density. The boxplot shows the median and the interquartile range, the whiskers cover minimal to maximal values. The white dot marks the mean. Data collected from a total of 40 cells of 4 independent experiments. Groups were tested for normality using the Shapiro–Wilk test and then compared using the Wilcoxon test. **p ≤ 0.01, ****p ≤ 0.0001.

Primary rat hippocampal neurons are widely used to analyze synaptic connections (Grabrucker et al., 2009). DIV 14 neurons provide a well-characterized maturation status (Figure 4A). After validating the pre-synaptic stainings, all nine SHANK3 antibodies were co-stained with SYNAPSIN or BASSOON, respectively (Figures 4B–J). The pictures of entire neurons are shown in Supplementary Figures 1A–I. In Figures 4B–J, a single neurite and the mask generated in ImageJ for analysis of the puncta are shown. To evaluate the synaptic performance of the SHANK3 antibodies, the number of puncta and the size of the puncta were analyzed. The puncta co-localizing with SYNAPSIN or BASSOON and the ones not co-localizing are shown. Comparing co-localizing and not co-localizing SHANK3 puncta, we expect that a specific SHANK3 antibody (1) recognizes more co-localizing than not co-localizing puncta, (2) does not give more than 40 puncta per 30 μm (Santuy et al., 2020) and (3) does not recognize structures that are far beyond the size of a synapse (Santuy et al., 2018). For Rock-GS (Figure 4B), SY-311 (Figure 4C), ASC (Figure 4F), and vNterm (Figure 4J) the numbers per 30 μm of dendrite reached up to 300 detected puncta, which would be an unphysiologically high synapse density (Santuy et al., 2020). Given that the antigen for the SY-311 is the same as for the SY-302 and SY-304, we can exclude the detection of different SHANK3 isoforms that could potentially show a different localization. For Rock-GS and ASC the antigen is unknown, opening the possibility for an isoform-specific recognition that does not localize to synapses. However, based on literature, it is unlikely, that solely non-synaptic isoforms would be recognized (Durand et al., 2012). In addition, either an increase of not co-localizing puncta compared to co-localizing ones or similar numbers in the two groups indicate, that the SHANK3-stained structures are not synapse specific. Fr1+2+3 (Figure 4I) presents a synaptic but also dendritic staining in between synapses. For SY-302 (Figure 4D), SY-304 (Figure 4E), CS (Figure 4G), and Fr1+2 (Figure 4H) a synaptic localization was observed. The number of SHANK3 puncta co-localizing with a pre-synaptic marker was significantly higher than the number of puncta, not co-localizing. Furthermore, the size of the co-localizing puncta was significantly bigger than of those not co-localizing. Intensity analysis supported these findings (Supplementary Figures 1A–I). The total intensity of SHANK3 puncta was significantly lower in the not co-localizing puncta than in the co-localizing ones for most of the antibodies. The average intensity as a ratio between total intensity and the puncta size was, according to the changes in each of the two parameters, not changed.

### Only Some SHANK3 Antibodies Give a Specific Synaptic SHANK3 Staining in Mouse Brain Sections

**FIGURE 5:**
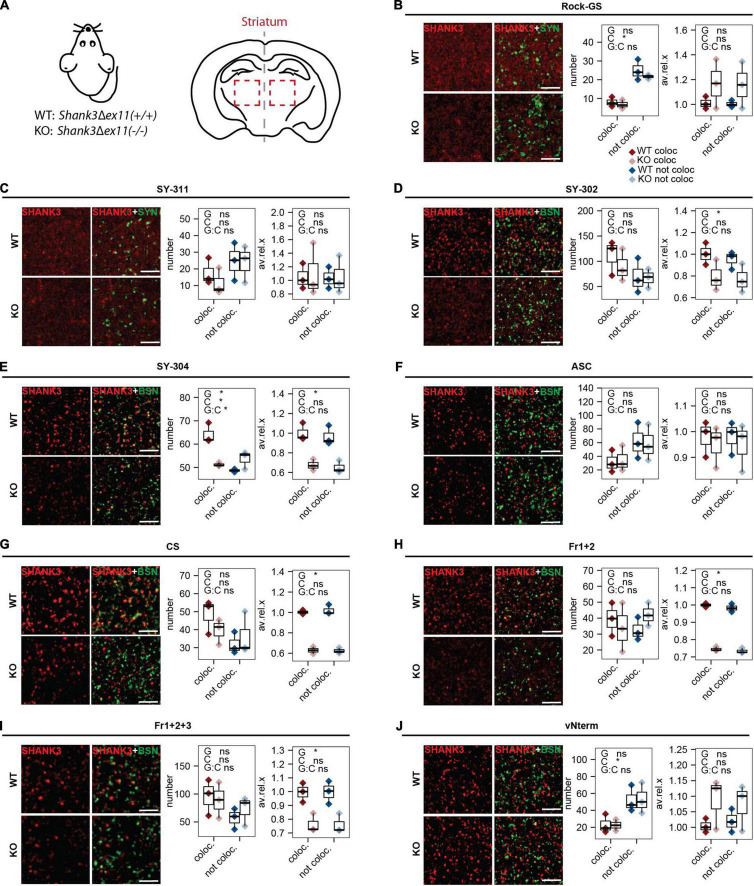
IHC analysis in striatum of mouse brain sections. **(A)** Sections of WT [*Shank3(*+*/*+*)*] and KO [*Shank3*Δ*ex11(–/–)*] mice were analyzed. Sections were stained against SYNAPSIN1/2 or BASSOON and **(B)** Rock-GS, **(C)** SY-311, **(D)** SY-302, **(E)** SY-304, **(F)** ASC, **(G)** CS, **(H)** Fr1+2, **(I)** Fr1+2+3, and **(J)** vNterm. Scale bar 5 μm. ICC of WT and KO are shown. Left graph: Number of synapses. SHANK3 puncta co-localizing with the respective pre-synaptic marker (red) or not co-localizing (blue) are shown. Right graph: Average intensity of SHANK3 puncta. The boxplot shows the median and the interquartile range, the whiskers cover minimal to maximal values. *n* = 3 animals per genotype. One-way ANOVA. G: genotype. C: co-localization or not. G:C: correlation of genotype and co-localization. *p ≤ 0.05.

In mouse brain sections, the striatum, which highly expresses SHANK3 (Monteiro and Feng, 2017), was analyzed in WT animals and *Shank3*Δ*ex11(–/–)* (KO; Figure 5A). As in the rat primary neurons, the SHANK3 puncta co-localizing with a pre-synaptic marker and the ones not co-localizing were compared. The statistical comparison of this contrast is indicated as “C” in each of the figures. The comparison of the two genotypes is indicated as “G.” We assessed the number of puncta and the puncta intensities (average gray value). The following antibodies gave a lower average gray value for the SHANK3 staining in KO compared to WT: SY-302 (Figure 5D), SY-304 (Figure 5E), CS (Figure 5G), Fr1+2 (Figure 5H), and Fr1+2+3 (Figure 5I), indicating that these antibodies can differentiate between the WT and the KO material. The numbers of puncta not co-localizing were higher than the ones co-localizing for Rock-GS (Figure 5B), SY-311 (Figure 5C), ASC (Figure 5F), and vNterm (Figure 5J), indicating that those SHANK3 antibodies either recognize more extra-synaptic than synaptic SHANK3 or are recognizing some other than SHANK3. Also, they did not show differences or gave even higher average intensities in the KO. Considering the total intensity, that takes the size of the puncta into consideration, the results followed those of the average intensity (Supplementary Figures 2A–I).

**FIGURE 6:**
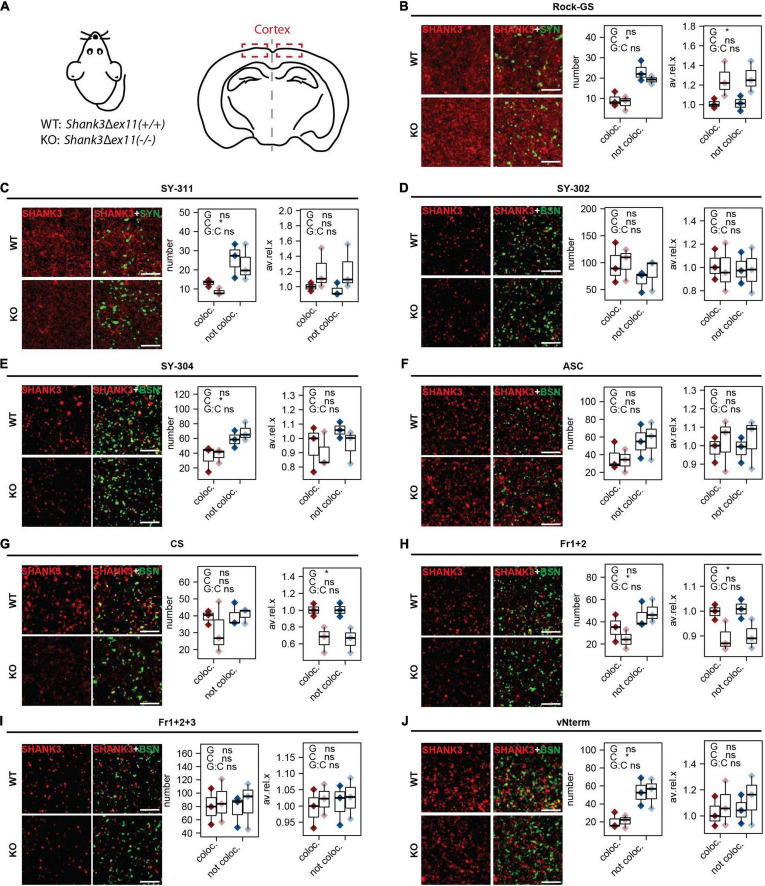
IHC analysis in cortex of mouse brain sections. **(A)** Sections of WT [*Shank3(*+*/*+*)*] and KO [*Shank3*Δ*ex11(–/–)*] mice were analyzed. Sections were stained against SYNAPSIN1/2 or BASSOON and **(B)** Rock-GS, **(C)** SY-311, **(D)** SY-302, **(E)** SY-304, **(F)** ASC, **(G)** CS, **(H)** Fr1+2, **(I)** Fr1+2+3, and **(J)** vNterm. Scale bar 5 μm. ICC of WT and KO are shown. Left graph: Number of synapses. SHANK3 puncta co-localizing with the respective pre-synaptic marker (red) or not co-localizing (blue) are shown. Right graph: Average intensity of SHANK3 puncta. The boxplot shows the median and the interquartile range, the whiskers cover minimal to maximal values. *n* = 3 animals per genotype. One-way ANOVA. G: genotype. C: co-localization or not. G:C: correlation of genotype and co-localization. *p ≤ 0.05.

The cortex was analyzed as a second region of interest (Figure 6A). The analysis was conducted the same way as for the striatum. The numbers of puncta not co-localizing were higher than the ones co-localizing for Rock-GS (Figure 6B), SY-311 (Figure 6C), SY-304 (Figure 6E), Fr1+2 (Figure 6H), and vNterm (Figure 5J). Only two antibodies did reveal significantly lower SHANK3 values in the KO compared to the WT, CS (Figure 6G), and Fr1+2 (Figure 6H). However, for both of them, the signal intensity was found lower in the KO in both co-localizing and not co-localizing puncta, indicating that also the extra-synaptic signal seems to be SHANK3 specific. The total intensity analysis, again, followed those results and the size of the puncta was not different (Supplementary Figures 3A–I).

### Comparison of SHANK3 Antibodies Used in This Study

**FIGURE 7:**
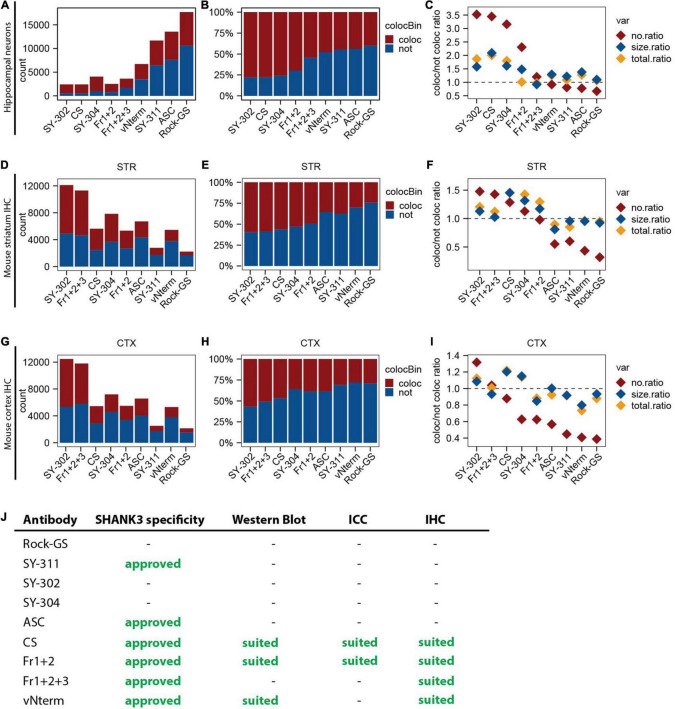
Summary of the results of the SHANK3 antibody validation. **(A)** Total count of all SHANK3 puncta detected in all primary hippocampal neurons. Co-localizing SHANK3 puncta with a pre-synaptic marker are depicted in red, not co-localizing ones in blue. **(B)** Relative count of all SHANK3 puncta detected in all primary hippocampal neurons in percentage. Co-localizing ones with a pre-synaptic marker are depicted in red, not co-localizing ones in blue. **(C)** Number (red), size (blue), and total intensity (yellow) of SHANK3 puncta in primary hippocampal neurons are depicted. For each of the variables the ratio between co-localizing and not co-localizing puncta was calculated. **(D)** Total count of all SHANK3 puncta detected in all WT striatal stainings. Co-localizing SHANK3 puncta with a pre-synaptic marker are depicted in red, not co-localizing ones in blue. **(E)** Relative count of all SHANK3 puncta detected in all WT striatal stainings. Co-localizing ones with a pre-synaptic marker are depicted in red, not co-localizing ones in blue. **(F)** Number (red), size (blue), and total intensity (yellow) of SHANK3 puncta in WT striatal stainings are depicted. For each of the variables the ratio between co-localizing and not co-localizing puncta was calculated. **(G)** Total count of all SHANK3 puncta detected in all WT cortical stainings. Co-localizing SHANK3 puncta with a pre-synaptic marker are depicted in red, not co-localizing ones in blue. **(H)** Relative count of all SHANK3 puncta detected in all WT cortical stainings. Co-localizing ones with a pre-synaptic marker are depicted in red, not co-localizing ones in blue. **(I)** Number (red), size (blue), and total intensity (yellow) of SHANK3 puncta in WT cortical stainings are depicted. For each of the variables the ratio between co-localizing and not co-localizing puncta was calculated. **(J)** Overview summarizing the performance of all SHANK3 antibodies tested in this study.

Bringing the results obtained from primary hippocampal neurons together, the synaptic density (count of puncta; Figure 7A) was compared. Since the same number of cells and the same lengths per dendrite have been used for all antibodies, they were summed up. The SHANK3 puncta co-localizing with a pre-synaptic marker and the not co-localizing ones are depicted in red and blue, respectively, and add up to the total number of puncta detected. The total number of puncta detected varies a lot between the antibodies. SY-302, CS, SY-304, Fr1+2, and Fr1+2+3 result in comparable numbers, while vNterm gives slightly higher numbers but especially SY-311, ASC, and Rock-GS show at least three times the amount. When calculating the relative amount of co-localizing and not co-localizing puncta by setting the count for each antibody to 100% (Figure 7B), we observed that for the antibodies detecting a reasonable number of puncta (SY-302, CS, SY-304, Fr1+2, and Fr1+2+3), approximately 75% of them were co-localizing with a pre-synaptic marker, while only approximately 30% of puncta were co-localizing for the SY-311, ASC, and Rock-GS.

To combine our findings in one graph, the ratio between co-localizing and not co-localizing puncta was built for the parameters number of puncta (no), displayed in red, size of puncta, displayed in blue, and the total intensity of puncta, displayed in yellow (Figure 7C). The higher the ratio, the more co-localizing and therefore synaptic puncta were detected. All ratios appeared to be above 1 for SY-302, CS, SY-304, and Fr1+2 stating them as the most promising antibodies for immunocytochemistry.

Following the same approach, the same graphs were generated for the mouse brain sections, only considering the WT stainings. For the striatum, the count of puncta (Figure 7D) gave us high total numbers of SY-302 and Fr1+2+3, intermediate numbers for CS, SY-304, Fr1+2, and vNterm, and low numbers for SY-311 and Rock-GS. Interestingly, the latter two are the ones giving not physiologically high numbers in immunocytochemistry. Considering the relative number of puncta in percentage (Figure 7E), most of the SHANK3 puncta were co-localizing with a pre-synaptic marker for SY-302, Fr1+2+3, CS, SY-304, and Fr1+2. For ASC, SY-311, vNterm, and Rock-GS, only a minority of puncta co-localized with a pre-synaptic marker. Combining all parameters and antibodies of the immunohistochemistry in the striatum in one graph (Figure 7F), the ratio between co-localizing and not co-localizing puncta was above one for number, size, and total intensity for SY-302, Fr1+2+3, CS, SY-304, and Fr1+2.

In cortex, the results for the total (Figure 7G) and relative count (Figure 7H) are almost the same as in the striatum, stating that the specificity in the striatum and the cortex is the same for these antibodies. When combining again all parameters in one graph (Figure 7I), only SY-302 and Fr1+2+3 reach a ratio above one for the parameters assessed.

To conclude for which method each of the tested antibodies is suited, we see the SHANK3 specificity addressed by the Western Blot analysis as a prerequisite to using the respective antibodies in any other application. Therefore, we only approve those antibodies for ICC and IHC being SHANK3 specific (Figure 7J). The SY-311 and the ASC antibodies are SHANK3-specific but did, in our hand, neither work in Western Blot analysis nor synaptic immunostainings. CS and Fr1+2 work well in Western Blot, ICC, and IHC. Fr1+2+3 can be used for IHC but not ICC and Western Blotting. The vNterm can be used in Western Blotting and IHC.

## Discussion

In this study, we tested nine different SHANK3 antibodies for their specificity and their suitability for Western Blotting, synaptic immunocyto-, and immunohistochemistry. The availability of commercially available antibodies directed against SHANK3 is of high interest for the fields, since they guarantee reproducibility of results also between different research groups. However, in our hands, most commercial SHANK3 antibodies included in this study did not pass our quality control.

We found that, in our hands, not all the antibodies are suited for all methods. With both the commercially available and our homemade antibodies, some should only be used for stainings and others only for Western Blotting. Based on our findings, we ask researchers to be careful when choosing their antibodies. The Rock-GS antibody is claimed to be suited for Western Blotting and IHC. In our hands, it passed the specificity test and worked in WB but not in IHC. The SY-311 is only suited for ICC according to the manufacturer but we did not obtain a reasonable SHANK3 staining pattern in ICC. The SY-302 and SY-304 should be considered with caution since they were not SHANK3-specific in our hands and are therefore not suited for any analysis, despite co-staining of several SHANK family members is desired. Interestingly, SY-311, SY-302, and SY-304 are all directed against the same epitope in SHANK3. Nevertheless, SY-311 is SHANK3-specific, while SY-302 and SY-304 are not. The CS antibody fulfilled all expectations. The Fr1+2 antibody can reliably be used in all applications tested. The Fr1+2+3 directed against an additional fragment within SHANK3 is SHANK3 specific and suited for IHC. The vNterm antibody is SHANK3 specific and worked well in WB and IHC. It is the only antibody of the study directed against the N-term of SHANK3, therefore giving a different isoform pattern than observed for the antibodies binding in the proline-rich region. Recently, other technologies for specific protein detection have become available, including aptamers and CRISPR/Cas-based protein detection, but these technologies are far less established and there are fewer available protein-specific products of these technologies to purchase. Also, well-characterized antibodies including the ones in this study can be used for comparison for surrogate technologies in the future.

An important finding of the study, is, in our opinion, the cross-reactivity of certain antibodies with SHANK1 and SHANK2. This issue can be addressed by overexpression experiments with SHANK1 and SHANK2 and WB analysis.

The combination of WB analysis of a tissue of interest together with IHC in KO material and co-localization analysis is widely accepted in the literature as a stringent control for antibody validation (Gautron, 2019). However, these authors also state that the validation experiments need to be executed properly and still do not guarantee specificity. They rise the possibility that a detailed validation process might not be necessary in the case of antibodies raised against a protein with a very well-known distribution pattern (Gautron, 2019). SHANK3 and its localization are indeed well known and have been studied intensively. Our results highlight that performing immunostaining and observing the desired localization of the signal, for example, a synaptic localization for SHANK3, is not enough to validate an antibody. Moreover, not even IHC with WT and KO tissue and a reduction of signal in KO can ensure specificity. With SY-311, -302, and -304 reduced signal intensities have been observed in the Shank3 KO tissue, but the antibodies are still not SHANK3-specific.

In this study, we focused on synaptic SHANK3 expression, but SHANK3 does not only localize to synapses but is also expressed in the nucleus (Grabrucker et al., 2014) and the cytoplasm (Wang et al., 2014). However, synaptic SHANK3 expression is best studied and offers a clear structure for analysis, as shown with different SHANK3 antibodies in the literature (Arons et al., 2012; Han et al., 2016; Kerrisk Campbell and Sheng, 2018; Wan et al., 2021). In respect of synaptic localization, the vNterm SHANK3 antibody can offer new insights. It has been reported that the overexpression of the SHANK3b isoform, which contains all N-terminal domains of SHANK3 including the PDZ domain, reduced the size and density of PSD-95 synaptic puncta and might therefore have more important functions in cellular specializations apart from the synapse (Wang et al., 2014). Therefore, the vNterm antibody might also detect non-synaptic SHANK3.

The beforementioned isoforms of SHANK3 add up to the complexity of subcellular SHANK3 localization and expression. SHANK3 isoforms are known to localize differentially within neurons (Wang et al., 2014). Different localization might, however, also predict potentially different functions, dependent on the protein domains that are expressed in the respective isoform. In regard to the antibodies used in this study, it is known that the SHANK3 recognized by our homemade antibodies does localize to the PSD. The homemade Fr1+2 antibody has its antigens in the proline-rich region (published earlier, Schmeisser et al., 2012). Given, that SY-311, SY-302, SY-304, and CS also have their antigen in the almost same part of the proline-rich region, we can assume that all of them should detect the same isoforms and therefore give a comparable staining pattern. The antibodies with unknown antigens cannot be interpreted in this way. The Rock-GS and the ASC antibodies give a dendritic but not synaptic staining. They might detect SHANK3 isoforms that are not synaptic and SHANK3 might also be transported in dendrites as it is known for axonal transport of SHANK3 (Han et al., 2016). The variety of different isoforms is therefore of great importance to interpret both the staining appearance of a SHANK3 antibody and also to understand distinct SHANK3 functions.

We used standard procedures; however, it could well be that certain antibodies would perform differentially with a different protocol. Several steps of the staining procedure can impact the performance of the antibodies, a fact that is often underestimated (True, 2008). The critical reagents for antibody performance in immunohistochemistry are the fixation, antigen retrieval, and the blocking reagent, but also the section thickness can influence the outcome. The fixation process is influenced by the type of fixative, the duration, temperature, and the pH while fixing the tissue (Leong, 2004). For our study, we performed PFA fixation and performed methanol fixation on selected antibodies in ICC, but this did not improve the staining outcome. Antigen retrieval is a highly sensitive process influenced by time, temperature, the pH, and the molarity of the retrieval solution (Leong, 2004). In our hands, antigen retrieval using citric acid has been performed in the IHC procedure, but the staining quality did not improve. Furthermore, we used different blocking reagents in both ICC and IHC, we varied the primary antibody dilution and the incubation period. In WB, different loading amounts have been tested, however, the most convincing methodology in our hand is that described in the Materials and Methods section. Indeed, in addition to finding the optimal protocols, the proper documentation of all steps of the analysis is critical for reproducible antibody performance, an issue that still is often neglected during the publication process (True, 2008; Jositsch et al., 2009).

Based on our findings in this study, we encourage authors and researchers in the field to validate the antibodies to help create a robust and reproducible methodology in SHANK research. This study provides one commercial and several homemade SHANK3 antibodies to bring this important point forward.

## Data Availability Statement

The raw data supporting the conclusions of this article will be made available by the authors, without undue reservation.

## Ethics Statement

The animal study was reviewed and approved by all animal experiments were performed in compliance with the guidelines for the welfare of experimental animals issued by the Federal Government of Germany and approved by the Regierungspräesidium Tüebingen and the local Ethics Committee at Ulm University; ID number: 1233.

## Author Contributions

A-KL, MS, and TB designed the project and experiments. A-KL and HFB performed the experiments and analyzed the data. VI performed the co-localization and statistical analysis. All authors contributed and wrote the manuscript.

## Conflict of Interest

The authors declare that the research was conducted in the absence of any commercial or financial relationships that could be construed as a potential conflict of interest.

## Publisher’s Note

All claims expressed in this article are solely those of the authors and do not necessarily represent those of their affiliated organizations, or those of the publisher, the editors and the reviewers. Any product that may be evaluated in this article, or claim that may be made by its manufacturer, is not guaranteed or endorsed by the publisher.

## Supplementary Material

Supplementary Figure 1 ICC in primary rat hippocampal neurons. A total cell is shown per staining (SHANK3 + SYNAPSIN1/2 or BSN). MAP2 staining is shown in white. **(A)** Rock-GS, **(B)** SY-311, **(C)** SY-302, **(D)** SY-304, **(E)** ASC, **(F)** CS, **(G)** Fr1+2, **(H)** Fr1+2+3, and **(I)** vNterm. Scale bar 20 μm. Left graph: Total intensity of the SHANK3 puncta. SHANK3 puncta co-localizing with the respective pre-synaptic marker (red) or not co-localizing (blue) are shown. Right graph: Average intensity of the SHANK3 puncta. The boxplot shows the median and the interquartile range, the whiskers cover minimal to maximal values. The white dot marks the mean. Data collected from a total of 40 cells of 4 independent experiments. Groups were tested for normality using the Shapiro-Wilk-Test and then compared using the Wilcoxon test. ^**^*p* ≤ 0.01, ^***^*p* ≤ 0.001, ^****^*p* ≤ 0.0001.

Supplementary Figure 2 Continued IHC analysis in striatum of mouse brain sections. Sections of WT [*Shank3(*+*/*+)] and KO [*Shank3*Δ*ex11(–/–)*] mice were analyzed. Sections were stained against SYNAPSIN1/2 or BASSOON and SHANK3. **(A)** Rock-GS, **(B)** SY-311, **(C)** SY-302, **(D)** SY-304, **(E)** ASC, **(F)** CS, **(G)** Fr1+2, **(H)** Fr1+2+3, and **(I)** vNterm. Left graph: Total intensity of SHANK3 puncta co-localizing with the respective pre-synaptic marker (red) or not co-localizing (blue) are shown. Right graph: Size of SHANK3 puncta. The boxplot shows the median and the interquartile range, the whiskers cover minimal to maximal values. *n* = 3 animals per genotype. One-way ANOVA. G: genotype. C: co-localization or not. G:C: correlation of genotype and co-localization. **p* ≤ 0.05.

Supplementary Figure 3 Continued IHC analysis in cortex of mouse brain sections. Sections of WT [*Shank3(*+*/*+*)*] and KO [*Shank3*Δ*ex11(–/–)*] mice were analyzed. Sections were stained against SYNAPSIN1/2 or BASSOON and SHANK3. **(A)** Rock-GS, **(B)** SY-311, **(C)** SY-302, **(D)** SY-304, **(E)** ASC, **(F)** CS, **(G)** Fr1+2, **(H)** Fr1+2+3, and **(I)** vNterm. Left graph: Total intensity of SHANK3 puncta co-localizing with the respective pre-synaptic marker (red) or not co-localizing (blue) are shown. Right graph: Size of SHANK3 puncta. The boxplot shows the median and the interquartile range, the whiskers cover minimal to maximal values. *n* = 3 animals per genotype. One-way ANOVA. G: genotype. C: co-localization or not. G:C: correlation of genotype and co-localization. **p* ≤ 0.05.

## References

[B1] Arons M. H. Thynne C. J. Grabrucker A. M. Li D. Schoen M. Cheyne J. E. (2012). Autism-associated mutations in ProSAP2/Shank3 impair synaptic transmission and neurexin-neuroligin-mediated transsynaptic signaling. *J. Neurosci.* 32 14966–14978. 10.1523/JNEUROSCI.2215-12.2012 23100419 PMC3752148

[B2] Boeckers T. M. Bockmann J. Kreutz M. R. Gundelfinger E. D. (2002). ProSAP/Shank proteins - a family of higher order organizing molecules of the postsynaptic density with an emerging role in human neurological disease. *J. Neurochem.* 81 903–910. 10.1046/j.1471-4159.2002.00931.x 12065602

[B3] Boeckers T. M. Liedtke T. Spilker C. Dresbach T. Bockmann J. Kreutz M. R. (2005). C-terminal synaptic targeting elements for postsynaptic density proteins ProSAP1/Shank2 and ProSAP2/Shank3. *J. Neurochem.* 92 519–524. 10.1111/j.1471-4159.2004.02910.x 15659222

[B4] Boeckers T. M. Winter C. Smalla K. H. Kreutz M. R. Bockmann J. Seidenbecher C. (1999). Proline-rich synapse-associated proteins ProSAP1 and ProSAP2 interact with synaptic proteins of the SAPAP/GKAP family. *Biochem. Biophys. Res. Commun.* 264 247–252. 10.1006/bbrc.1999.1489 10527873

[B5] Delling J. P. Boeckers T. M. (2021). Comparison of SHANK3 deficiency in animal models: phenotypes, treatment strategies, and translational implications. *J. Neurodev. Disord.* 13:55. 10.1186/s11689-021-09397-8 34784886 PMC8594088

[B6] Durand C. M. Betancur C. Boeckers T. M. Bockmann J. Chaste P. Fauchereau F. (2007). Mutations in the gene encoding the synaptic scaffolding protein SHANK3 are associated with autism spectrum disorders. *Nat. Genet.* 39 25–27. 10.1038/ng1933 17173049 PMC2082049

[B7] Durand C. M. Perroy J. Loll F. Perrais D. Fagni L. Bourgeron T. (2012). SHANK3 mutations identified in autism lead to modification of dendritic spine morphology via an actin-dependent mechanism. *Mol. Psychiatry* 17 71–84. 10.1038/mp.2011.57 21606927 PMC3252613

[B8] Gautron L. (2019). On the Necessity of Validating Antibodies in the Immunohistochemistry Literature. *Front. Neuroanat.* 13:46. 10.3389/fnana.2019.00046 31080409 PMC6497795

[B9] Grabrucker A. Vaida B. Bockmann J. Boeckers T. M. (2009). Synaptogenesis of hippocampal neurons in primary cell culture. *Cell Tissue Res.* 338 333–341. 10.1007/s00441-009-0881-z 19885679

[B10] Grabrucker A. M. Knight M. J. Proepper C. Bockmann J. Joubert M. Rowan M. (2011a). Concerted action of zinc and ProSAP/Shank in synaptogenesis and synapse maturation. *EMBO J.* 30 569–581. 10.1038/emboj.2010.336 21217644 PMC3034012

[B11] Grabrucker A. M. Schmeisser M. J. Schoen M. Boeckers T. M. (2011b). Postsynaptic ProSAP/Shank scaffolds in the cross-hair of synaptopathies. *Trends Cell Biol.* 21 594–603. 10.1016/j.tcb.2011.07.003 21840719

[B12] Grabrucker S. Proepper C. Mangus K. Eckert M. Chhabra R. Schmeisser M. J. (2014). The PSD protein ProSAP2/Shank3 displays synapto-nuclear shuttling which is deregulated in a schizophrenia-associated mutation. *Exp. Neurol.* 253 126–137. 10.1016/j.expneurol.2013.12.015 24382453

[B13] Han Q. Kim Y. H. Wang X. Liu D. Zhang Z. J. Bey A. L. (2016). SHANK3 Deficiency Impairs Heat Hyperalgesia and TRPV1 Signaling in Primary Sensory Neurons. *Neuron* 92 1279–1293. 10.1016/j.neuron.2016.11.007 27916453 PMC5182147

[B14] Herbert A. D. Carr A. M. Hoffmann E. (2014). FindFoci: a focus detection algorithm with automated parameter training that closely matches human assignments, reduces human inconsistencies and increases speed of analysis. *PLoS One* 9:e114749. 10.1371/journal.pone.0114749 25478967 PMC4257716

[B15] Jiang Y. H. Ehlers M. D. (2013). Modeling autism by SHANK gene mutations in mice. *Neuron* 78 8–27. 10.1016/j.neuron.2013.03.016 23583105 PMC3659167

[B16] Jositsch G. Papadakis T. Haberberger R. V. Wolff M. Wess J. Kummer W. (2009). Suitability of muscarinic acetylcholine receptor antibodies for immunohistochemistry evaluated on tissue sections of receptor gene-deficient mice. *Naunyn Schmiedebergs Arch. Pharmacol.* 379 389–395. 10.1007/s00210-008-0365-9 18974978 PMC3896859

[B17] Kerrisk Campbell M. Sheng M. (2018). USP8 Deubiquitinates SHANK3 to Control Synapse Density and SHANK3 Activity-Dependent Protein Levels. *J. Neurosci.* 38 5289–5301. 10.1523/JNEUROSCI.3305-17.2018 29735556 PMC6596000

[B18] Leong A. S. (2004). Pitfalls in diagnostic immunohistology. *Adv. Anat. Pathol.* 11 86–93. 10.1097/00125480-200403000-00002 15090844

[B19] Lutz A. K. Pfaender S. Incearap B. Ioannidis V. Ottonelli I. Fohr K. J. (2020). Autism-associated SHANK3 mutations impair maturation of neuromuscular junctions and striated muscles. *Sci. Transl. Med.* 12:eaaz3267. 10.1126/scitranslmed.aaz3267 32522805

[B20] Monteiro P. Feng G. (2017). SHANK proteins: roles at the synapse and in autism spectrum disorder. *Nat. Rev. Neurosci.* 18 147–157. 10.1038/nrn.2016.183 28179641

[B21] Naisbitt S. Kim E. Tu J. C. Xiao B. Sala C. Valtschanoff J. (1999). Shank, a novel family of postsynaptic density proteins that binds to the NMDA receptor/PSD-95/GKAP complex and cortactin. *Neuron* 23 569–582. 10.1016/s0896-6273(00)80809-0 10433268

[B22] Pfaender S. Sauer A. K. Hagmeyer S. Mangus K. Linta L. Liebau S. (2017). Zinc deficiency and low enterocyte zinc transporter expression in human patients with autism related mutations in SHANK3. *Sci. Rep.* 7:45190. 10.1038/srep45190 28345660 PMC5366950

[B23] Phelan K. Boccuto L. Powell C. M. Boeckers T. M. van Ravenswaaij-Arts C. Rogers R. C. (2022). Phelan-McDermid syndrome: a classification system after 30 years of experience. *Orphanet. J. Rare Dis.* 17:27. 10.1186/s13023-022-02180-5 35093143 PMC8800328

[B24] Phelan K. McDermid H. E. (2012). The 22q13.3 Deletion Syndrome (Phelan-McDermid Syndrome). *Mol. Syndromol.* 2 186–201. 22670140 10.1159/000334260 PMC3366702

[B25] Phelan M. C. (2008). Deletion 22q13.3 syndrome. *Orphanet. J. Rare Dis.* 3:14. 10.1186/1750-1172-3-14 18505557 PMC2427010

[B26] Romorini S. Piccoli G. Jiang M. Grossano P. Tonna N. Passafaro M. (2004). A functional role of postsynaptic density-95-guanylate kinase-associated protein complex in regulating Shank assembly and stability to synapses. *J. Neurosci.* 24 9391–9404. 10.1523/JNEUROSCI.3314-04.2004 15496675 PMC6730104

[B27] Sala C. Piech V. Wilson N. R. Passafaro M. Liu G. Sheng M. (2001). Regulation of dendritic spine morphology and synaptic function by Shank and Homer. *Neuron* 31 115–130. 10.1016/s0896-6273(01)00339-7 11498055

[B28] Santuy A. Rodriguez J. R. DeFelipe J. Merchan-Perez A. (2018). Study of the Size and Shape of Synapses in the Juvenile Rat Somatosensory Cortex with 3D Electron Microscopy. *eNeuro* 5:ENEURO.0377-17.2017. 10.1523/ENEURO.0377-17.2017 29387782 PMC5790755

[B29] Santuy A. Tomas-Roca L. Rodriguez J. R. Gonzalez-Soriano J. Zhu F. Qiu Z. (2020). Estimation of the number of synapses in the hippocampus and brain-wide by volume electron microscopy and genetic labeling. *Sci. Rep.* 10:14014. 10.1038/s41598-020-70859-5 32814795 PMC7438319

[B30] Sauer A. K. Bockmann J. Steinestel K. Boeckers T. M. Grabrucker A. M. (2019). Altered Intestinal Morphology and Microbiota Composition in the Autism Spectrum Disorders Associated SHANK3 Mouse Model. *Int. J. Mol. Sci.* 20:2134. 10.3390/ijms20092134 31052177 PMC6540607

[B31] Schmeisser M. J. Ey E. Wegener S. Bockmann J. Stempel A. V. Kuebler A. (2012). Autistic-like behaviours and hyperactivity in mice lacking ProSAP1/Shank2. *Nature* 486 256–260. 10.1038/nature11015 22699619

[B32] Shcheglovitov A. Shcheglovitova O. Yazawa M. Portmann T. Shu R. Sebastiano V. (2013). SHANK3 and IGF1 restore synaptic deficits in neurons from 22q13 deletion syndrome patients. *Nature* 503 267–271. 10.1038/nature12618 24132240 PMC5559273

[B33] True L. D. (2008). Quality control in molecular immunohistochemistry. *Histochem. Cell Biol.* 130 473–480. 10.1007/s00418-008-0481-0 18648842 PMC2522330

[B34] Tu J. C. Xiao B. Naisbitt S. Yuan J. P. Petralia R. S. Brakeman P. (1999). Coupling of mGluR/Homer and PSD-95 complexes by the Shank family of postsynaptic density proteins. *Neuron* 23 583–592. 10.1016/s0896-6273(00)80810-7 10433269

[B35] Verpelli C. Dvoretskova E. Vicidomini C. Rossi F. Chiappalone M. Schoen M. (2011). Importance of Shank3 protein in regulating metabotropic glutamate receptor 5 (mGluR5) expression and signaling at synapses. *J. Biol. Chem.* 286 34839–34850. 10.1074/jbc.M111.258384 21795692 PMC3186429

[B36] Wan L. Ai J. Q. Yang C. Jiang J. Zhang Q. L. Luo Z. H. (2021). Expression of the Excitatory Postsynaptic Scaffolding Protein, Shank3, in Human Brain: effect of Age and Alzheimer’s Disease. *Front. Aging Neurosci.* 13:717263. 10.3389/fnagi.2021.717263 34504419 PMC8421777

[B37] Wang X. Xu Q. Bey A. L. Lee Y. Jiang Y. H. (2014). Transcriptional and functional complexity of Shank3 provides a molecular framework to understand the phenotypic heterogeneity of SHANK3 causing autism and Shank3 mutant mice. *Mol. Autism* 5:30. 10.1186/2040-2392-5-30 25071925 PMC4113141

