# Supplementary figures and images for: SHANK3 Antibody Validation: Differential Performance in Western Blotting, Immunocyto- and Immunohistochemistry
Source: Front Synaptic Neurosci. 2022 Jun 6;14:890231. doi: 10.3389/fnsyn.2022.890231 (PMC9207774; doi:10.3389/fnsyn.2022.890231)

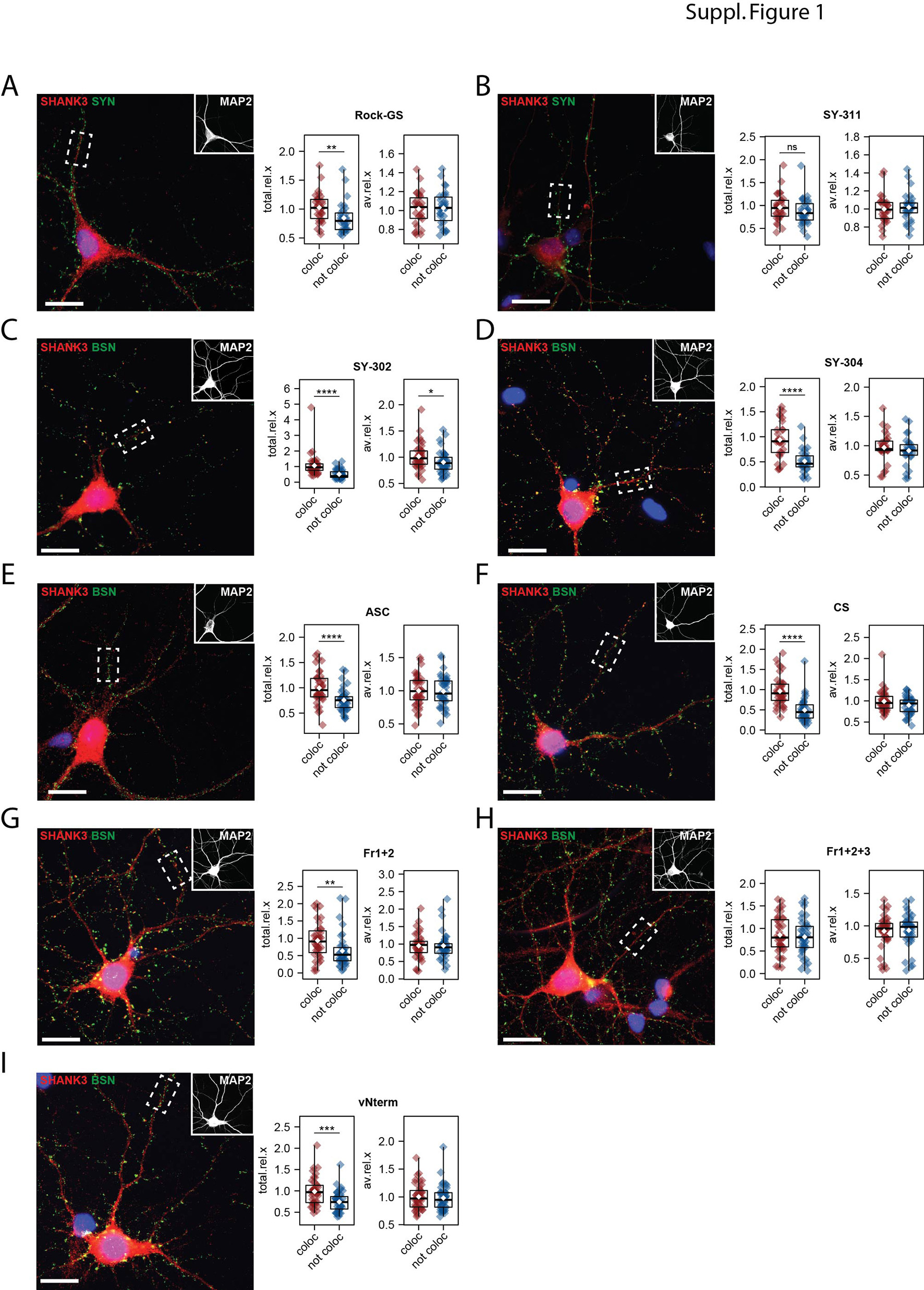

Supplement: Supplementary Figure 1 — ICC in primary rat hippocampal neurons. A total cell is shown per staining (SHANK3 + SYNAPSIN1/2 or BSN). MAP2 staining is shown in white. (A) Rock-GS, (B) SY-311, (C) SY-302, (D) SY-304, (E) ASC, (F) CS, (G) Fr1+2, (H) Fr1+2+3, and (I) vNterm. Scale bar 20 μm. Left graph: Total intensity of the SHANK3 puncta. SHANK3 puncta co-localizing with the respective pre-synaptic marker (red) or not co-localizing (blue) are shown. Right graph: Average intensity of the SHANK3 puncta. The boxplot shows the median and the interquartile range, the whiskers cover minimal to maximal values. The white dot marks the mean. Data collected from a total of 40 cells of 4 independent experiments. Groups were tested for normality using the Shapiro-Wilk-Test and then compared using the Wilcoxon test. **p ≤ 0.01, ***p ≤ 0.001, ****p ≤ 0.0001. [file Image_1.JPEG]

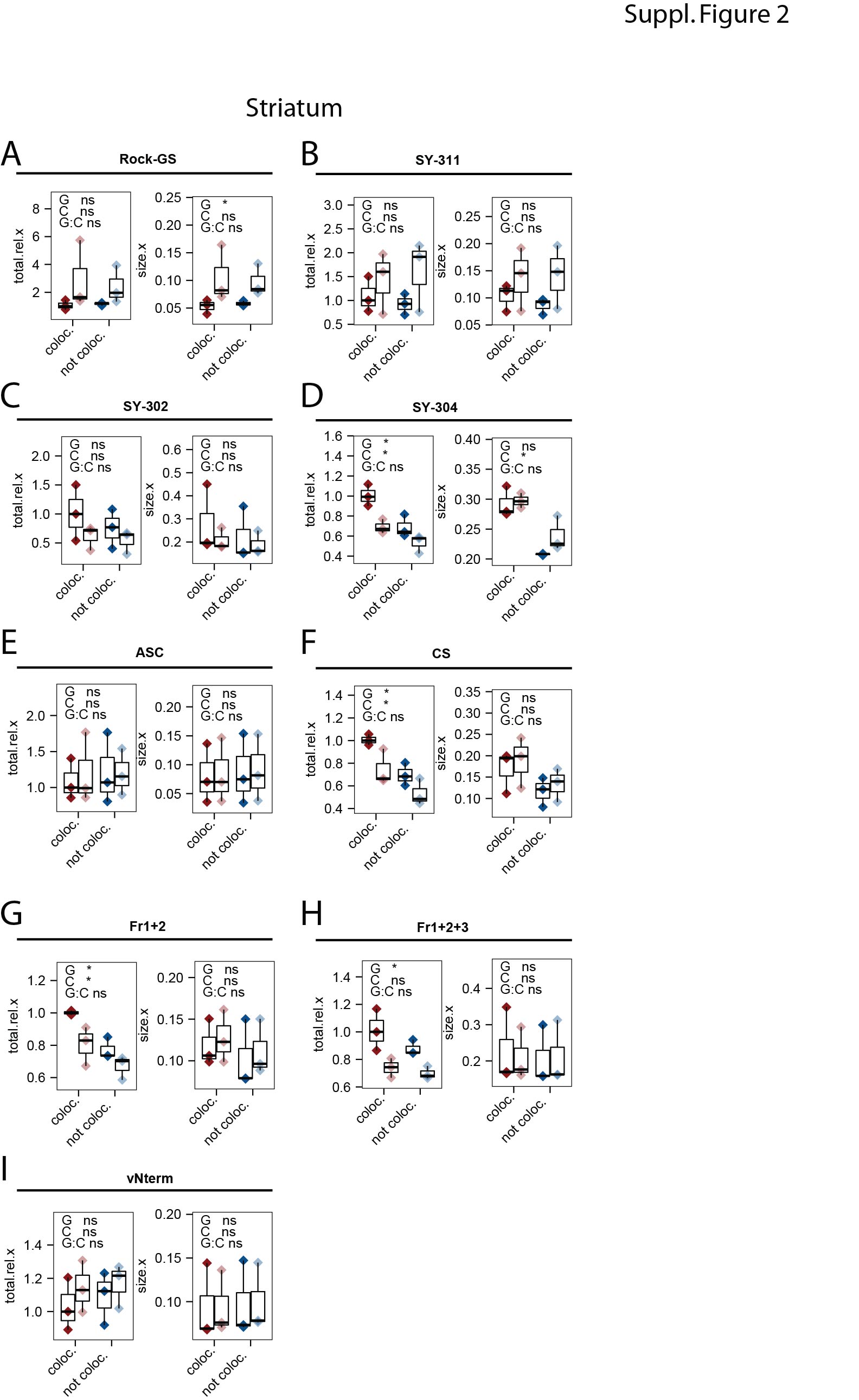

Supplement: Supplementary Figure 2 — Continued IHC analysis in striatum of mouse brain sections. Sections of WT [Shank3(+/+)] and KO [Shank3Δex11(–/–)] mice were analyzed. Sections were stained against SYNAPSIN1/2 or BASSOON and SHANK3. (A) Rock-GS, (B) SY-311, (C) SY-302, (D) SY-304, (E) ASC, (F) CS, (G) Fr1+2, (H) Fr1+2+3, and (I) vNterm. Left graph: Total intensity of SHANK3 puncta co-localizing with the respective pre-synaptic marker (red) or not co-localizing (blue) are shown. Right graph: Size of SHANK3 puncta. The boxplot shows the median and the interquartile range, the whiskers cover minimal to maximal values. n = 3 animals per genotype. One-way ANOVA. G: genotype. C: co-localization or not. G:C: correlation of genotype and co-localization. *p ≤ 0.05. [file Image_2.JPEG]

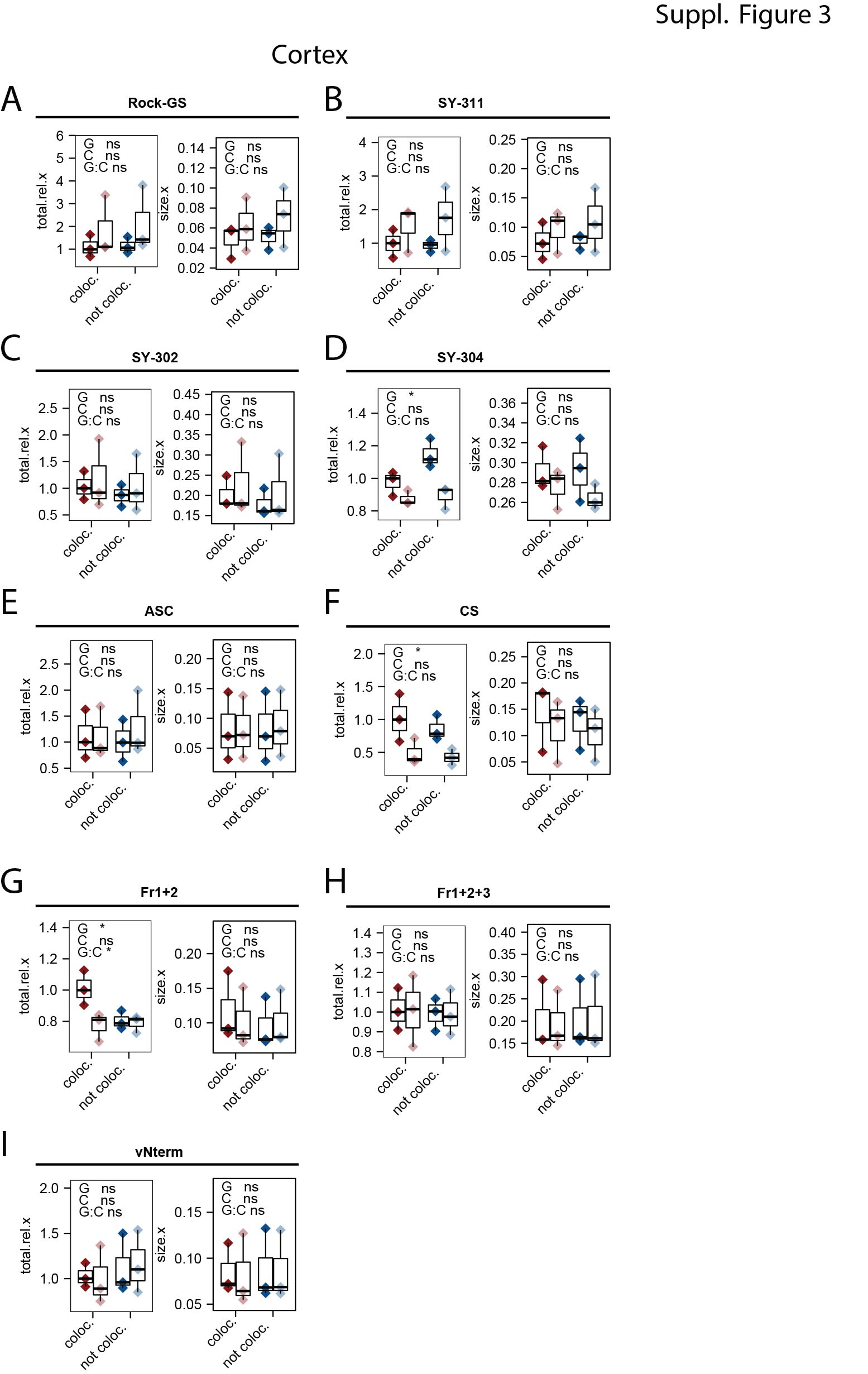

Supplement: Supplementary Figure 3 — Continued IHC analysis in cortex of mouse brain sections. Sections of WT [Shank3(+/+)] and KO [Shank3Δex11(–/–)] mice were analyzed. Sections were stained against SYNAPSIN1/2 or BASSOON and SHANK3. (A) Rock-GS, (B) SY-311, (C) SY-302, (D) SY-304, (E) ASC, (F) CS, (G) Fr1+2, (H) Fr1+2+3, and (I) vNterm. Left graph: Total intensity of SHANK3 puncta co-localizing with the respective pre-synaptic marker (red) or not co-localizing (blue) are shown. Right graph: Size of SHANK3 puncta. The boxplot shows the median and the interquartile range, the whiskers cover minimal to maximal values. n = 3 animals per genotype. One-way ANOVA. G: genotype. C: co-localization or not. G:C: correlation of genotype and co-localization. *p ≤ 0.05. [file Image_3.JPEG]
